# The Coxsackievirus and Adenovirus Receptor Has a Short Half-Life in Epithelial Cells

**DOI:** 10.3390/pathogens11020173

**Published:** 2022-01-27

**Authors:** Poornima Kotha Lakshmi Narayan, James M. Readler, Mahmoud S. Alghamri, Trisha L. Brockman, Ran Yan, Priyanka Sharma, Vladislav Snitsarev, Katherine J. D. A. Excoffon, Abimbola O. Kolawole

**Affiliations:** 1 Department of Biological Sciences, Wright State University, Dayton, OH 45435, USA; poornimakotha@gmail.com (P.K.L.N.); readler.3@wright.edu (J.M.R.); alghamma@med.umich.edu (M.S.A.); trisha.brockman.20@gmail.com (T.L.B.); renaranyan@gmail.com (R.Y.); priyankasharma18@gmail.com (P.S.); katherine.excoffon@wright.edu (K.J.D.A.E.); 2 Biomedical Sciences PhD Program, Wright State University, Dayton, OH 45435, USA; 3 Biology Department, Montclair State University, Montclair, NJ 07043, USA; Snitsarevv@mail.montclair.edu

**Keywords:** human adenovirus, coxsackievirus and adenovirus receptor, half-life, polarized epithelia

## Abstract

The coxsackievirus and adenovirus receptor (CAR) is an essential cellular protein that is involved in cell adhesion, cell signaling, and viral infection. The 8-exon encoded isoform (CAR^Ex8^) resides at the apical surface of polarized epithelia, where it is accessible as a receptor for adenovirus entering the airway lumen. Given its pivotal role in viral infection, it is a target for antiviral strategies. To understand the regulation of CAR^Ex8^ and determine the feasibility of receptor downregulation, the half-life of total and apical localized CAR^Ex8^ was determined and correlated with adenovirus transduction. Total and apical CAR^Ex8^ has a relatively short half-life of approximately 2 h. The half-life of apical CAR^Ex8^ correlates well with adenovirus transduction. These results suggest that antiviral strategies that aim to degrade the primary receptor for apical adenovirus infection will be effective within a relatively short time frame after application.

Acute respiratory tract infections are a major cause of morbidity and mortality worldwide, especially among children and elderly populations. In particular, respiratory viruses are one of the major contributors of acute respiratory tract infections [1,2]. Human adenoviruses (HAdV) mediate respiratory infections that are usually self-limiting in healthy individuals. However, these viruses can occasionally cause fatal illness in both adults and children, especially in immunosuppressed populations [3]. Novel approaches to inhibit HAdV infection in both healthy and susceptible populations are likely to reduce the morbidity and mortality associated with HAdV pathogenesis. HAdV are classified into seven species (HAdV-A through -G) and include more than 100 genotypes (http://hadvwg.gmu.edu/122021) [4]. Like all viruses, the first step for successful HAdV infection is attachment to the host cell. All species, except HAdV-B, can use the coxsackievirus and adenovirus receptor (CAR) as the primary receptor for attachment [5,6,7]. Two transmembrane isoforms of CAR are present in polarized epithelial cells: an isoform encoded by the first seven exons (CAR^Ex7^) of the *CXADR* gene and an isoform that is alternatively spliced from a cryptic splice site within the seventh exon to the eighth exon (CAR^Ex8^) [8,9]. As a result of this differential splicing event, CAR^Ex7^ and CAR^Ex8^ differ only in the amino acid sequence at the extreme C-terminus. This difference is responsible for the differential localization of the two CAR isoforms within polarized epithelial cells. CAR^Ex7^, the more abundant isoform, localizes at the basolateral surface of polarized epithelial cells, while CAR^Ex8^ localizes at the apical surface. Our lab has previously shown that CAR^Ex8^ mediates apical adenoviral infection in polarized epithelial cells and that the susceptibility to adenovirus infection correlates with the level of CAR^Ex8^ expression [8,10,11,12,13,14]. Moreover, HAdV appears to have co-opted an innate immune response that stimulates the expression and the localization of CAR^Ex8^ at the apical surface of polarized epithelial cells in order to more efficiently gain entry and initiate infection [13].

CAR has many important physiologic functions, including: homophilic and heterophilic cellular adhesion, immune activation, and cardiac conduction [15,16,17,18,19,20,21]. CAR knockout is embryonically lethal as it is crucial for normal development of the heart and lymphatic system [21,22,23,24]. CAR expression is shown to correlate with some types of epithelial cell-derived cancer, such as lung, breast, ovary, and cervical cancers [18,25,26,27,28,29,30]. In polarized epithelial cells, CAR^Ex7^ forms homodimers on the basolateral surface with those on the adjacent cells and is essential for maintaining the epithelial cell junctions and barrier integrity [31,32,33,34]. We also discovered a novel function for CAR^Ex8^, which is to facilitate the innate immune response by serving as an adhesion protein for infiltrating neutrophils at the apical surface of polarized epithelial cells [13]. Given the physiologic importance of CAR^Ex8^, it is logical to assume that regulatory mechanisms tightly control its expression. Therefore, the goal of these studies was to determine the half-life of CAR^Ex8^ and correlate the expression levels at the apical surface with adenovirus infection for a better understanding of CAR^Ex8^ regulation.

The generation of MDCK cell lines stably carrying a doxycycline (Dox)-inducible expression cassette for FLAG-tagged human CAR^Ex8^ has previously been described in detail [13]. In order to determine the half-life of CAR^Ex8^ in the inducible epithelial cells, we first asked how long it takes to detect CAR^Ex8^ expression after exposure to Dox. To address this question, MDCK-CAR^Ex8^ stable cells were seeded in a plastic 12-well dish in FBS tet^(−)^ media. The cells were induced with 100 ng/mL Dox for 24 h, and then harvested at the indicated time points. Cell lysates were analyzed for FLAG-tagged CAR^Ex8^ protein expression via Western blot (WB) as previously described [10]. CAR^Ex8^ could be detected as early as 4 h after induction with Dox and increased over time (Figure 1A). The removal of Dox causes the translation of FLAG-tagged human CAR^Ex8^ to cease, allowing for the measurement of CAR^Ex8^ turnover. The results show that the induced CAR^Ex8^ protein degraded over time and was undetectable after 24 h. Next, using a pulse chase experimental design, we again monitored CAR^Ex8^ protein degradation over time. MDCK-CAR^Ex8^-stable cells were seeded in a plastic 12-well dish in FBS tet^(−)^ media and CAR^Ex8^ expression was induced with 100 ng/mL Dox. At 24 h post induction, Dox was removed and the cells were harvested at the indicated time points and analyzed by WB. CAR^Ex8^ expression was stable for approximately 8 h after Dox removal, after which the protein level decreased rapidly, by half at 10 h. CAR^Ex8^ decreased again by another half from 10 h to 12 h and was undetectable at 24 h (Figure 1B). The WB bands were quantified and normalized to actin using ImageJ in order to calculate the half-life of CAR^Ex8^. The half-life of CAR^Ex8^ was determined to be 2.0 ± 1.4 h (Figure 1C). As the primary adenovirus receptor, the presence of CAR^Ex8^ at the apical surface, where it is accessible to incoming virions, is arguably the major factor that defines the susceptibility of the epithelium to viral infection. To determine the rate of CAR^Ex8^ turnover at the apical surface, we induced polarized MDCK-CAR^Ex8^ epithelia with media containing 100 ng/mL Dox for 24 h, as previously described [13]. After 24 h of induction, Dox was removed and replaced with fresh tet^(−)^ medium for 0, 4, 8, 12 and 24 h. The treatment with Dox and removal of Dox was staggered so that surface biotinylation for all conditions could be performed at the same time. Once each time point was met, apical surface biotinylation was performed as described [35], and the biotinylated proteins were isolated with streptavidin beads and analyzed via WB. The CAR^Ex8^ protein appears to be relatively stable for the initial 4 h after Dox removal but then decrease to undetectable levels by 24 h. By 8 h post Dox removal, the CAR^Ex8^ protein appears to have decreased to nearly 25% of its original amount (Figure 1D). A quantification of the CAR^Ex8^ protein bands revealed that the half-life of apical CAR^Ex8^ is like that of a total CAR^Ex8^ at 1.8 ± 1.9 h (Figure 1E).

We conducted these studies in the MDCK epithelial cell line because of its extensive use in studies related to cell polarity [13,35,36]. These cells form well-defined junctions, are easy to cultivate, and are suitable for both 2D and 3D cultures [37]. However, the CAR^Ex8^ half-life in an inducible MDCK stable cell line may present several limitations: (i) Lung and kidney epithelial cells may degrade proteins using different mechanisms or at different rates; (ii) The gene that encodes FLAG-tagged CAR^Ex8^ within the integrated inducible vector is human [38]. Thus, the species difference may result in differential recognition or degradation for endogenous CAR; (iii) Overexpression of this protein may overwhelm the cell’s regulatory machinery; (iv) Residual mRNA from overexpression or Dox remaining within the cell may allow for continued protein translation despite the removal of Dox from the media, possibly altering the time frame of degradation; and (v) The FLAG-tag on the protein may disturb or alter the rate of protein degradation. To address these limitations and confirm that the MDCK-CAR^Ex8^-stable cell line was an appropriate model system, the half-life of endogenous CAR^Ex8^ was also determined in the Calu-3 cell line, a well-studied human lung adenocarcinoma cell line able to polarize into an epithelium. This was accomplished by inhibiting protein synthesis in Calu-3 cells with 30 μg/mL cycloheximide treatment for increasing periods of time prior to epithelial cell lysis. The epithelia were then lysed and the total cellular levels of CAR^Ex8^ were measured by WB using CAR^Ex8^-specific Ab (5678p, University of Iowa), relative to actin (Figure 2A). The half-life of 2.4 h ± 0.05 h obtained for endogenous CAR^Ex8^ in Calu-3 cells was similar to the half-life found for CAR^Ex8^ in the MDCK-stable cell lines (Figure 2B).

The induction of MDCK CAR^Ex8^-stable cells with Dox increased the expression of CAR^Ex8^ (Figure 1) and correspondingly increased the adenovirus infection of polarized epithelial cells [13]. The removal of Dox reverted the cells back to a state in which no FLAG-tagged CAR^Ex8^ could be detected (Figure 1), and background levels of CAR^Ex8^ were very low [13,39]. Thus, we hypothesized that the susceptibility of the MDCK epithelium to apical HAdV5 transduction would decrease at the same rate as CAR^Ex8^ degradation. To test this, polarized MDCK CAR^Ex8^-stable cells were induced with 100 ng/mL Dox for 24 h. After 24 h of induction, Dox was removed and replaced with fresh tet^(−)^ medium for 0, 4, 6, 8, 10, 12 and 24 h. The treatment with Dox and removal of Dox were staggered so that the β-galactosidase assay for all conditions could be performed at the same time. Once each time point was met, the apical surfaces of epithelia were inoculated with a recombinant adenovirus carrying the β-galactosidase reporter gene (HAdV5-β-Gal; MOI 100, University of Iowa Vector Core, Iowa City, IA, USA) for 1 h, as previously described [13]. The apical surface was then washed, and β-galactosidase activity was analyzed 24 h later to determine AdV transduction as described [35]. The results show a significant decrease in AdV5 transduction (Figure 3) that corresponds well with the decrease in CAR^Ex8^ protein levels (Figure 1). These results indicate that CAR^Ex8^ localized at the apical surface of polarized epithelia acts as the primary receptor for adenoviruses. Our data also confirm that the presence of apical CAR^Ex8^ is one of the primary factors determining the susceptibility of a polarized epithelium to HAdV infection [13].

Overall, this work highlights the potential importance of targeting CAR^Ex8^ as a means of preventing HAdV infection [14]. Protein turnover plays an important role in all cellular processes. Therefore, knowing the rate at which a protein is degraded can have significant implications for the advancement of directed therapeutics in treating many diseases. In instances where there are several different isoforms of a protein, with the differential localization of each separate isoform, and a varied amount of each isoform under standard cellular conditions, knowing the half-life of each isoform could provide an insight into protein function and regulation. Protein degradation is usually a first-order reaction; thus, the half-lives of CAR^Ex8^ in different cell lines were analyzed using a plateau followed by a one-phase exponential decay [40,41]. Protein half-lives differ from protein to protein. In mammalian cells, the global median protein half-life is ~46 h [42]. The localization of a protein within a polarized epithelium may play a significant role in a protein’s half-life. Apical proteins have been shown to have half-lives ranging from ~1 h to ~24 h and global analysis conferred that plasma membrane proteins can have half-lives of 100 h or shorter half-lives of <4 h [43,44,45,46]. Our data showed that the half-life curves contained a plateau followed by a one-phase decay. The plateau principle is applied to biological systems when a drug is being administered continuously or at steady intervals, which can affect cellular functions. It also applies to endogenous cases, such as protein abundance in response to a hormone. In our case, our cells were exposed to a continuous amount of Dox for a 24 h time frame. Dox induced the expression of mRNA transcripts. Once the Dox was removed, we hypothesized that the plateau effect was caused by residual mRNA being translated after Dox removal. Additionally, CAR^Ex8^ was shown to be negatively regulated by the scaffolding protein MAGI-1 [10,47]. This could account for decreased protein stability, and thus a relatively short half-life. The short half-life of CAR^Ex8^ may also be a consequence of the protein’s native physiological role in neutrophil transmigration, a process that only requires CAR^Ex8^ to be expressed in short, transient bursts.

In summary, our findings demonstrate that apical CAR^Ex8^ has a half-life of approximately 2 h and that the residence of CAR^Ex8^ at the apical surface is a major factor in determining the susceptibility of an epithelium to HAdV infection. These data suggest that novel methods to enhance the downregulation of the primary apical adenovirus receptor, such as targeting MAGI-1, would work in a reasonably short time frame and, similarly, be reversible in a relatively short time frame [14]. This may lead to a novel class of antivirals that decrease the apical viral infection of epithelia.

## Figures and Tables

**Figure 1:**
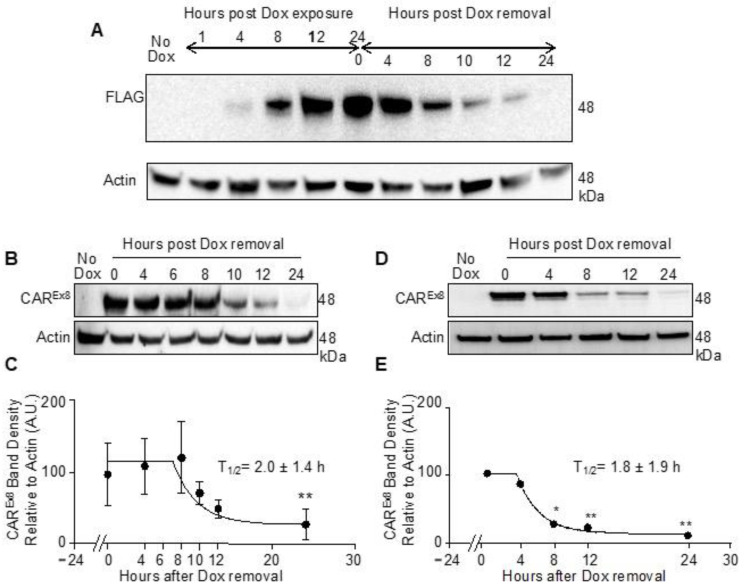
CAR^Ex8^ has a half-life of approximately 2 h. MDCK cells that stably expressed FLAG-tagged CAR^Ex8^ under the control of a doxycycline-sensitive promoter were seeded in a plastic 12-well dish in FBS tet^(−)^ media. Cells were induced with 100 ng/mL doxycycline for 24 h. The doxycycline-containing media were then removed and replaced with standard FBS tet^(−)^ culture media. (**A**). Cells were lysed at indicated time points before and after removal of Dox and the lysates were analyzed by Western blot (WB) using anti-FLAG or actin-specific primary antibodies. (**B**). Cells were lysed at indicated time points post Dox removal and lysates were analyzed by WB. (**C**). CAR^Ex8^ bands were normalized to their corresponding actin bands and quantified using ImageJ. (**D**). MDCK-FLAG-CAR^Ex8^ were polarized on semipermeable membranes. At 24 h post Dox treatment, standard FBS tet^(−)^ culture media were added and surface biotinylation was performed at indicated time points post Dox removal. Cells were then lysed and the lysates (actin) or isolated-biotinylated proteins (CAR^Ex8^, FLAG Ab) were analyzed via WB. (**E**). CAR^Ex8^ bands were normalized to their corresponding actin bands and quantified using ImageJ. Error bars represent SEM. (* *p* < 0.05; ** *p* < 0.01; One-Way ANOVA; N at least 3).

**Figure 2:**
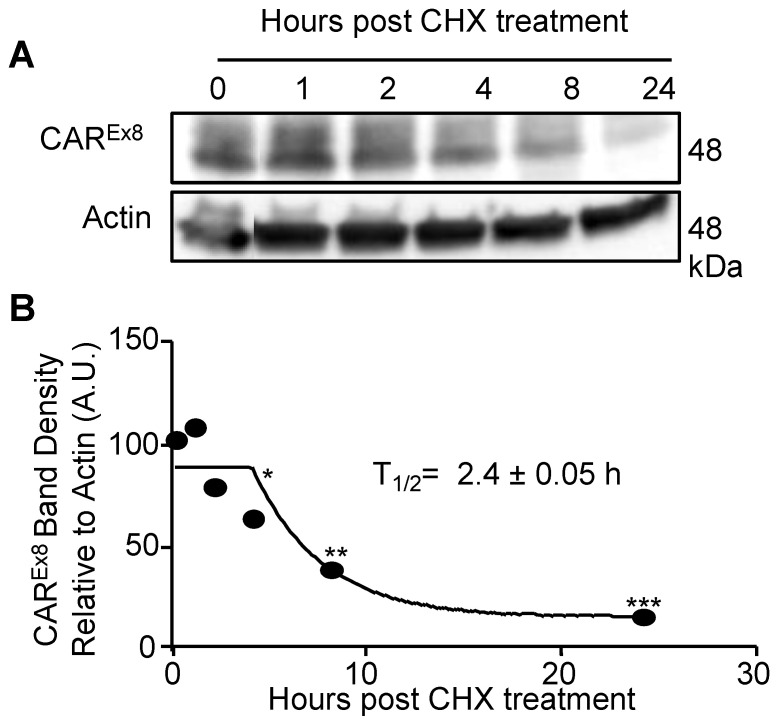
CAR^Ex8^ has a half-life of approximately 2 h. (**A**) Calu-3 cells were treated with 30 μg/mL cycloheximide. The cells were lysed at the times indicated post cycloheximide addition and analyzed by WB using a CAR^Ex8^-specific primary antibody (5678p) or actin (loading control). (**B**) Quantification of CAR^Ex8^ bands normalized to actin. Error bars represent SEM. (* *p* < 0.05; ** *p* < 0.01; *** *p* < 0.001; One-Way ANOVA; N at least 3).

**Figure 3:**
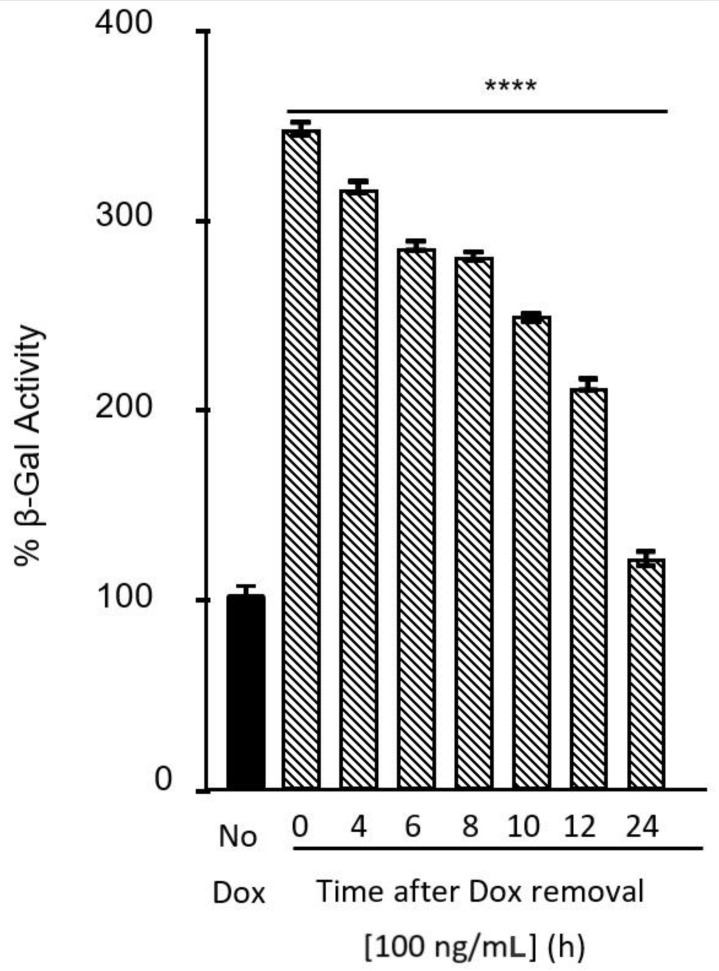
Adenovirus transduction decreases as CAR^Ex8^ degrades. MDCK-FLAG-CAR^Ex8^ were seeded and polarized in FBS tet^(−)^ media. The cells were then treated with 100 ng/mL Dox for 24 h. At 24 h post treatment, the doxycycline-containing media were removed and replaced with standard FBS tet^(−)^ culture media. At 4, 6, 8, 10, 12, and 24 h post dox removal, cells were transduced with AdV-β-Gal. AdV transduction was analyzed by β-galactosidase assay after 24 h. Error bars indicate standard error of the mean. (**** *p* < 0.0001; One-Way ANOVA; N = 3).
